# Utility of Native T1 mapping to differentiate between athlete's heart and non-ischemic dilated cardiomyopathy

**DOI:** 10.1186/1532-429X-17-S1-P379

**Published:** 2015-02-03

**Authors:** Ify Mordi, David Carrick, Hiram Bezerra, Nikolaos Tzemos

**Affiliations:** 1 Institute of Cardiovascular and Medical Sciences, University of Glasgow, Glasgow, UK; 2 University Hospitals Case Medical Center, Case Western Reserve University, Cleveland, OH, USA

## Background

CMR has become an increasingly valuable tool in the diagnosis and risk stratification of patients with non-ischaemic dilated cardiomyopathy (DCM) due to its assessment of left ventricular systolic function and tissue characterization ability and may have a role in early identification of cardiomyopathy. Some of the changes associated with early DCM (left ventricular dilatation and mild reduction in LV ejection fraction) can also occur in patients with a history of significant aerobic exercise, known as "athlete's heart". Using standard echocardiographic and CMR parameters it may be difficult to differentiate between DCM and normal physiological athletic adaptation, which may have significant implications for the patient. We hypothesized that use of CMR tagging and T1 and T2 mapping might be useful to differentiate between patients with left ventricular dilatation due to DCM and athlete's heart.

## Methods

105 male patients (27 healthy controls, 41 patients with severe DCM - LVEF <35%, 16 sedentary patients and 21 athletes (both with LVEF 45-55%) underwent a comprehensive CMR protocol including tagging, T1 and T2 mapping and calculation of extracellular volume (ECV) using a 1.5T scanner.

## Results

GCS was significantly decreased in both athletes and sedentary patients with compared to controls (GCS -12.87 ± 3.61% vs. -13.05 ± 4.37% vs. -16.53 ± 2.01%, p<0.001 respectively) while there was a trend to lower GLS in athletes compared to controls. Native T1 and ECV were significantly increased in sedentary mild DCM patients compared to both healthy controls (native T1 1017 ± 42ms vs. 952 ± 31ms, p<0.001; ECV 31.2 ± 4.1% vs. 26.2 ± 2.8%, p=0.003) and athletes (native T1 1017 ± 42ms vs. 958 ± 30ms, p<0.001; ECV 31.2 ± 4.1% vs. 26.4 ± 6.8%, p=0.004). Using multivariable logistic regression native T1 was the only significant parameter to differentiate between athletes and sedentary patients with early DCM (AUC 0.91).

## Conclusions

T1 mapping is potentially a useful tool for differentiating between athlete's heart and patients with early DCM. T1 mapping could be used whenever differentiation between these two phenotypes is inconclusive using alternative techniques.

## Funding

N/A.

**Figure 1:**
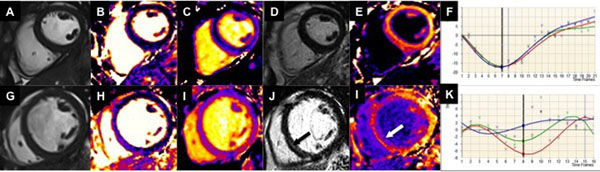
**Examples of the protocol for a healthy control (top) and patient with severe DCM (bottom).** Top: A 52 year-old healthy male control with a normal sized left ventricle (end-diastolic diameter 5.6 cm) and preserved left ventricular ejection fraction (57.8%) (A). Septal T2 was 51.6 ms (B), septal native T1 924.9 ms (C) and there was no LGE (D) or significant diffuse fibrosis (post-contrast T1 467.1 ms, ECV 27%, E). GCS was -15.68% (F). Bottom: A 58 year-old severe DCM patient with a dilated left ventricle (end-diastolic diameter 8.0 cm) and severely impaired left ventricular ejection fraction (28.4%) (G). Septal T2 was 59.8 ms (H), septal native T1 1017.8 ms (I). There was midwall LGE (J; arrow) also reflected by the reduced post-contrast T1 and ECV (post-contrast T1 429.8 ms, ECV 35%, I; arrow). GCS was -5.15% (K).

**Figure 2:**
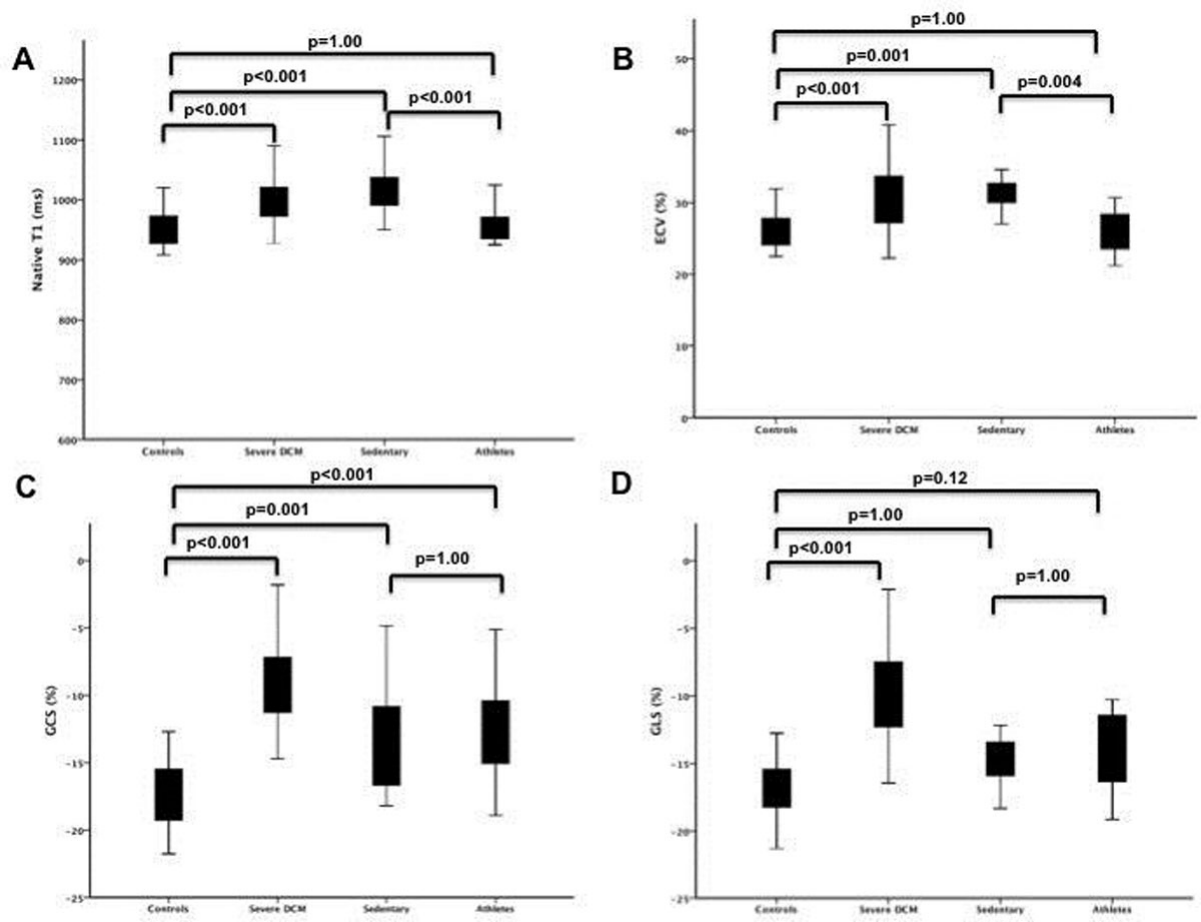
**Comparison between controls, patients with severe DCM, sedentary patients with mild DCM and athletes.** Comparison between the four groups using native T1 (A), ECV (B), GCS (C) and GLS (D).

